# Swimming‐related effects on cerebrovascular and cognitive function

**DOI:** 10.14814/phy2.14247

**Published:** 2019-10-22

**Authors:** Leena N. Shoemaker, Luke C. Wilson, Samuel J. E. Lucas, Liana Machado, Kate N. Thomas, James D. Cotter

**Affiliations:** ^1^ School of Physical Education, Sport and Exercise Sciences University of Otago Dunedin New Zealand; ^2^ Department of Medicine Dunedin School of Medicine University of Otago Dunedin New Zealand; ^3^ Department of Psychology University of Otago Dunedin New Zealand; ^4^ Department of Physiology University of Otago Dunedin New Zealand; ^5^ School of Sport, Exercise and Rehabilitation Sciences College of Life and Environmental Sciences University of Birmingham Birmingham UK; ^6^ Centre for Human Brain Health University of Birmingham Birmingham UK; ^7^ Department of Surgical Sciences Dunedin School of Medicine University of Otago Dunedin New Zealand

**Keywords:** Cerebral blood flow, cognition, reaction time, swimming, water immersion

## Abstract

Both acute and regular exercise influence vascular and cognitive function. Upright aquatic exercise increases mean middle cerebral artery blood velocity (MCAv_mean_) and has been suggested as favorable for cerebrovascular adaptations. However, MCAv_mean_ has not been reported during swimming. Thus, we examined the cerebrovascular and cognitive effects of swimming. Ten land‐based athletes (22 ± 5 years) and eight swimmers (19 ± 1 years) completed three cognitive tasks and four conditions that were used to independently and collectively delineate the swimming‐related factors (i.e., posture, immersion, CO_2_ retention [end‐tidal CO_2_; PETCO_2_], and motor involvement). Measurements of MCAv_mean_ and PETCO_2_ were taken throughout each condition. Prone posture increased MCAv_mean_ by 11% (*P* < 0.01 vs. upright land). Water immersion independently increased MCAv_mean_ when upright (12%; *P* < 0.01) but not prone (*P* = 0.76). The consequent rise in PETCO_2_ during head‐out, breast‐stroke swimming (50% heart rate range) independently increased MCAv_mean_ by 14% (*P* < 0.01), while the motor involvement of swimming per se did not significantly change MCAv_mean_ (*P* = 0.32). While accounting for sex, swimmers had ~17% lower MCAv_mean_ during all rest conditions (*P* ≤ 0.05). However, in a subset of participants, both groups had similar internal carotid artery diameters (*P* = 0.99) and velocities (*P* = 0.97). Water immersion per se did not alter cognition (*P* ≥ 0.15), but 20 min of moderate‐intensity swimming improved visuomotor performance by 4% (*P* = 0.03), regardless of athlete group (*P* = 0.12). In conclusion, breast‐stroke swimming increased MCAv_mean_ mostly due to postural and PETCO_2_ effects, with minimal contributions from water immersion or motor activity. Lastly, swimming improved cognitive functioning acutely, regardless of athlete group. Future research should explore the chronic effects of swimming on cerebrovascular function and cognition, particularly in aging.

## Introduction

Exercise improves cognition both acutely and chronically (Hillman et al., 2008). It has been proposed that the cognitive benefit from exercise may be associated with the acute, exercise‐related increase in cerebral blood flow (Ogoh, 2017). Leg‐based dynamic exercise, such as cycling (Jorgensen et al., 1992; Hellstrom et al., 1996; Ogoh and Ainslie, 2009), recumbent stepping (Billinger et al., 2017), and treadmill running (Jiang et al., 1995) increase cerebral blood velocity (CBV) through the independent and interactive factors involved in cerebral blood flow regulation (e.g., blood pressure [BP], cardiac output, and alterations in arterial CO_2_; Ide and Secher, 2000, Ogoh and Ainslie, 2009). Furthermore, upright leg‐based, aquatic exercise has recently been shown to augment the increase in CBV compared to intensity‐matched land exercise (Parfitt et al., 2017).

Water immersion itself has numerous pronounced cardiorespiratory effects that directly influence cerebral perfusion control. For example, hydrostatic pressure increases stroke volume and mean arterial pressure (MAP) despite the counteractive decrease in heart rate (HR). These cardiac‐related factors, in combination with immersion‐associated CO_2_ retention may be the cause of increased CBV during water immersion (Carter et al., 2014; Pugh et al., 2014). Additional factors different from land‐based or other water‐based exercise, may further influence cerebral perfusion control; for example, prone posture, hydrostatic‐related changes in systolic BP and MAP, intrinsic and extrinsic influences on stroke volume, and increased neurovascular coupling due to increased somatosensory and motor involvement. However, while swimming presents a unique stimulus for cerebral perfusion in regard to regulatory mechanisms, its cerebrovascular effects have not been examined in either acute or chronic (i.e., training‐specificity) contexts. Nor have the separate and combined effects of these factors on cerebral perfusion been examined.

Cognition – a key and complex role of the brain – benefits from acute bouts of exercise (Chang et al., 2012). Acutely, reaction time appears to be improved after 20‐min durations (Brisswalter et al., 2002) of moderate‐intensity exercise (McMorris and Hale, 2012). Although there are data supporting the cognitive‐benefit of swim *training* in older individuals (Hawkins et al., 1992; Abou‐Dest et al., 2012; Vasegowda, 2018), there remains little information for young adults, particularly when measured in an acute setting. Furthermore, it remains unclear if the cognitive‐benefit of acute exercise is affected by training‐specificity (Pesce, 2009; Lambourne and Tomporowski, 2010).

Therefore, the primary aim of this study was to determine the cerebrovascular response to moderate‐intensity swimming and identify the independent and combined effects of: (1) posture, (2) water immersion, (3) CO_2_ retention, and (4) motor involvement. A secondary aim was to examine the acute effects of swimming and changes in CBV on different aspects of cognition. We examined these questions in 18 people who were regularly physically active – almost half of whom were trained swimmers. Based on previous literature, performance on a multi‐level reaction time task was measured before and after 20 min of moderate‐intensity breast‐stoke swimming.

## Methods

### Ethical approval

This study was approved by the University of Otago Human Ethics committee (H16/143) and conducted in accordance with the standards set by the Declaration of Helsinki. Written and informed consent was obtained from all participants prior to any data collection.

### Participants

Ten land‐based recreational athletes and eight swimmers volunteered for the study. Participant characteristics are illustrated in Table 1. One female participant was in her follicular phase of menstrual cycle, with the remaining eight in luteal phase or on oral contraception (active pill phase). All participants were non‐smokers and apparently free from neurological, cerebro‐ and cardiovascular, or psychological disorders. Participants reported to the laboratory being well‐hydrated and having abstained from strenuous exercise and alcohol for 12 h, and caffeine, food and moderate exercise for a minimum of 2 h. All participants reported being moderately to very physically active. Majority of participants (15/18) had a $\dot{V}O_{2\text{max}}$ within or above the 80^th^ percentile, as determined by ACSM aerobic fitness classifications by age and sex (American College of Sports Medicine et al., 2018). The remaining three were above the 50th (2/18) and 75th (1/18) percentiles for their age and sex group.

**Table 1:** Participant characteristics.

	Land‐based athletes	Swimmers
Sample size	10 (6 female)	8 (3 female)
Age (y)	22 ± 5	19 ± 1
Height (cm)	175 ± 6	179 ± 10
Mass (kg)	68 ± 5	76 ± 10
$\dot{V}O_{2\text{max}}$ (mL/min/kg)	54 ± 6	56 ± 10
Swimming 50% HRR (bpm)	130 ± 8	132 ± 9
ICA diameter (cm)	4.9 ± 0.5	4.9 ± 0.6
ICA velocity (cm/s)	39.8 ± 8.7	39.6 ± 10.0

Mean ± SD; Between group differences all *P* ≥ 0.09; $\dot{V}O_{2\text{max}}$, maximal rate of oxygen consumed; HRR, heart rate range; ICA, internal carotid artery (*N* = 8 [3 male] and 5 [3 male] for land‐based athletes and swimmers, respectively).

### Protocol overview and design

**Figure 1:**
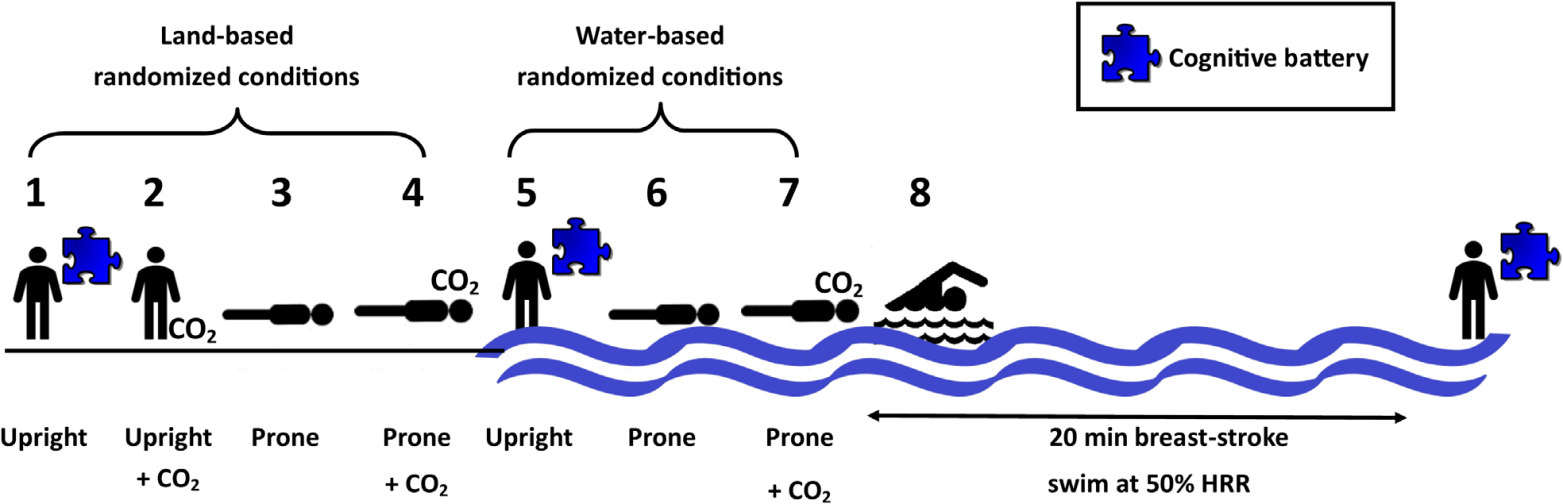
Protocol schematic of eight conditions used to characterize the separate and combined effects of the swimming‐related factors on cerebral blood flow. The order of conditions on land (1–4) and in water (5–7) were randomized across participants. Participants completed a cognitive battery (indicated by a puzzle piece) on land and in water before and after a 20 min, moderate‐intensity breast‐stroke swim. The +CO_2_ represents a CO_2_ stimulus to match each individual's 50% HRR swimming poikilo‐capnia obtained during the familiarization session. HRR, heart rate range.

All testing was completed in the swimming flume and adjacent laboratory at the School of Physical Education, Sport and Exercise Sciences (University of Otago, Dunedin, NZ). Each participant first completed a familiarization session, followed by an experimental session wherein the swimming‐related effects on CBV were delineated via eight conditions (Fig. 1). The familiarization and experimental sessions occurred at similar times of day within participants. Between groups, an equal number of sessions occurred in the morning (6:30 to 11:30 am), afternoon (12:00 to 4:30 pm), and evening (5:00 to 8:30 pm).

### Familiarization

This session familiarized all participants to the protocol and equipment and obtained individual values for maximal oxygen consumption ($\dot{V}O_{2\text{max}}$) and heart rate range (HRR). Upon instrumentation, $\dot{V}O_{2\text{max}}$ was first determined from an incremental running test. HR and breath‐by‐breath gas analysis (Quark CPET, COSMED, Italy) were recorded during a step‐incremental maximal exertion protocol. A successful test was determined by at least two of the following criteria from Edvardsen et al. (2014): HR ≥85% of age‐predicted maximum (220 − age), a respiratory exchange ratio value of ≥1.10, a plateau in $\dot{V}O_{2}$ (<150 mL/min) despite an increase in workload, and a failure to maintain the workload.

As HR is lower in water than on land for the same exercise (Parfitt et al., 2017), a maximal swim test was undertaken 45 min after the treadmill $\dot{V}O_{2\text{max}}$ test, to determine each participant’s swimming‐specific HRR. After being briefly familiarized to head‐out breast‐stroke technique and instructed to breathe as normal for swimming, participants swam head‐out breast‐stroke for incremental 2‐min stages, until exhaustion. Maximal swimming velocities ranged from 0.5 to 1.3 m/s. Partial pressures of end‐tidal CO_2_ (PETCO_2_) and HR were recorded during this test, and the value (averaged across 30 s) at 50% HRR was used during the following experimental session. Due to the complexity of the breast‐stroke swimming style, participants were offered further instruction and practice after recovery from the max test. However, only two participants requested further instruction. Before and after these two fitness tests, participants practiced a cognitive battery, described below.

### Experimental session

The second session assessed the independent and combined effects of the 4 swimming‐related factors that could affect cerebrovascular function; (1) posture (i.e., prone vs. upright), (2) hydrostatic pressure (i.e., water immersion), (3) increased PETCO_2_, and (4) motor involvement (i.e., breast‐stroke swimming,). The postures were compared on land and with water immersion, with (+8 ± 4 mm Hg) and without increased PETCO_2_. The PETCO_2_ level was matched to the individual’s PETCO_2_ values whist swimming at 50% of HRR from the max test during the familiarization session. Each of eight conditions (Fig. 1) lasted a minimum of 5 min, with additional time between conditions to allow for recovery. The CO_2_ wash‐in period was a minimum of 1 min and wash‐out was a minimum of 5 min, depending on randomization. To confidently report steady‐state physiological measures, data were collected (i.e., MAP, PETCO_2_, etc.) and allocated for analysis after a minimum of 3 min in each condition. All four land conditions occurred in randomized order, as did the first three water‐immersed conditions. Land conditions always occurred prior to any water immersion. This was to avoid potential reductions in core temperature which would have been caused by enhanced heat offload due to participants being wet following water immersion, and associated temperature and wet clothes/skin discomfort.

A 20 min, moderate‐intensity, head‐up breast‐stroke swim was maintained at 50% HRR, with water velocity adjusted to accommodate the drift in HR. Cognitive testing (lasting ~2 min) was completed while participants stood in an upright posture on land and in water before and immediately after the 20‐min swim.

### Water immersion

Pilot testing confirmed the flume water temperature to be controlled at 30°C (within a range of 28–30°C), as a compromise between being cold stressful during rest and heat stressful during sustained swimming. While in an upright posture, immersion depth was controlled at approximately the level of the right atrium. For the prone posture with water immersion, participants were prone with their head up and arms extended beyond their head, with buoyancy aids facilitating a passive streamlined position.

### Physiological testing

Cerebral blood velocity in the left middle cerebral artery (MCAv) was continuously measured using a 2‐MHz pulsed transcranial Doppler ultrasound (Spencer Doppler, Redmond, WA). The probe was secured with a headband device to maintain insonation angle and position (Spencer Doppler) on the transtemporal window of the skull, above the zygomatic arch. Depth of insonation was set between 40 and 55 mm. Mean MCAv (MCAv_mean_) was calculated as mean time integrals (cm/s/s) and divided by cardiac period (s:derived from the MCAv waveform). MCAv pulsatility index was calculated as end‐diastolic velocity subtracted from peak systolic velocity and divided by MCAv_mean_.

Heart rate was derived from the MCAv waveform. BP was taken during each condition with an automated BP cuff with tubing extension (OMRON HEM‐7322, OMRON Healthcare CO. Ltd, Kyoto, Japan). MAP is reported as the weighted systolic (1/3) and diastolic (2/3), averaged across 3 repeated measurements. Cerebrovascular conductance was calculated as MCAv_mean_/MAP.

For all land‐based $\dot{V}O_{2\text{max}}$ tests, participants breathed through a leak‐free respiratory mask (Hans‐Rudolf 8980, Kansas City, MO) attached to a turbine (Quark CPET, COSMED, Italy). For all maximal breast‐stroke swimming tests, PETCO_2_ was measured continuously using a modified 3‐inch snorkel, with a respiratory gas sample line and an online gas analyzer (Model CD‐3A Carbon Dioxide Analyzer, AEI Technologies, Bastrop, TX). A customized dynamic end‐tidal clamping system (C.E.T. Gas Clamp, School of Physical Education, Sport and Exercise Sciences, University of Otago, Dunedin, NZ) accurately controlled PETCO_2_ within 2 min and 2 mm Hg using a two‐way non‐rebreathing valve and leak‐free respiratory mask. Hypercapnia (to match individual poikilo‐capnia during 50% HRR swimming) was achieved and maintained by addition of dry CO_2_ gas into room air using a solenoid‐valve control. In this instance, no compensatory control of oxygen was required as end‐tidal oxygen never dropped below 100 mm Hg. Cerebrovascular hypercapnic reactivity (CVR_CO2_) was calculated using linear regression as the change in MCAv_mean_ divided by the change in mean PETCO_2_ (cm/s/mm Hg). All data were sampled continuously using an analog‐to‐digital converter (1000 Hz; Powerlab /16SP ML795; ADInstruments, Dunedin, NZ) and stored for offline analysis on Labchart software (version 7, ADInstruments).

### Cognitive testing

The cognitive battery was designed to measure basic visuomotor, inhibition, and mental switching performance (White et al., 2017). This 2‐min battery contains three tasks that assess different domains of cognitive control: (1) Pro trials measure basic visuomotor performance; (2) Anti trials measure inhibitory control; and (3) Pro/Anti trials measure mental switching. Briefly, participants saw a red or green stimulus positioned to the left or right of a central fixation point and were instructed to press the corresponding button as quickly as possible, without sacrificing accuracy. To account for speed‐accuracy trade off, accuracy‐adjusted reaction time (aRT; median correct reaction time/(1 − error rate)) was calculated for each of the three cognitive tasks. In unpublished work we have found that aRT is a reliable measure across time with intraclass correlation coefficients of 0.92, 0.86 and 0.92 for Pro, Anti, and Pro/Anti respectively, when comparing two trials 2 h apart (*n* = 25).

### Carotid ultrasound

To validate an initial finding (discussed below), 13 available participants (five swimmers and eight land‐based athletes; three males in each group) attended the laboratory for a brief follow‐up measurement of their internal carotid artery (ICA) diameter and velocity. Measurements were in supine posture, using ultrasound (Terason T3300, Teratech, Burlington, MA) with a 15 MHz linear array transducer by simultaneously recording a longitudinal section B‐mode image of the artery and a spectral Doppler trace of blood velocity. Measurements were made ~2 cm distal to the carotid bifurcation according to published guidelines (Thomas et al., 2015). Screen recording software (Camtasia Studio 8, TechSmith, Okemos, MI) captured the screen in a video file for later offline analysis. Wall tracking software (Cardiovascular Suite v 3.5.3, Quipu, Pisa, Italy) was used to determine a 1‐min average of diameter and velocity. Test–retest reliability (coefficient of variation) for this operator (K. T.) using this software for diameter and velocity were 0.4% and 2.1% respectively (*n* = 10).

### Statistical analysis

Statistics for confirmation of normality and changes in primary outcome variables were calculated using IBM SPSS (Version 25.0 for Windows, IBM Corporation, Armonk, NY) and graphed with GraphPad Prism (Prism Version 7, GraphPad Software, CA). Student *t*‐tests were used to compare baseline values between groups and compare the relevant physiological outcomes during 50% HRR swimming to baseline. MCAv_mean_ was assessed using four separate mixed 2‐way repeated measures (RM) analyses of covariance (ANCOVA), which allowed for the analysis of group effects (2 levels; swimmers vs. land‐based athletes) with each of the following: posture (2 levels; prone vs. upright), prone water immersion (2 levels; land vs. water), PETCO_2_ (2 levels; prone in water with vs. without clamped PETCO_2_), or swimming (2 levels; passive streamline position with raised PETCO_2_ vs. active swimming), while controlling for sex. Sex was included as a covariate due to the sex‐proportion differences between groups, and because it has been shown that females have higher baseline MCAv_mean_ than males (e.g., Peltonen et al., 2015). Similarly, four 2‐way RM analysis of variances (ANOVA) were used to analyze the effects of posture, water immersion, PETCO_2_, and swimming for secondary physiological outcomes such as MAP, HR, and PETCO_2_. Additionally, two 2‐way RM ANOVAs were run to consider cognitive performance (i.e., Pro, Anti, and Pro/Anti aRT) in relation to group and upright water immersion (2 levels; land vs. water) or swimming (2 levels; pre‐ vs. post‐swim). An α of 0.05 was used for each ANCOVA and ANOVA. Data are reported as mean ± standard deviations. Effect sizes are reported as partial eta‐squares (ηp^2^) and can be interpreted as small (≤0.01), medium (0.06), or large (≥0.14) effects (Cohen, 1977; Richardson, 2011).

## Results

All 18 participants successfully completed the original protocol requirements. Due to availability, only 13 participants completed the additional carotid ultrasound.

**Figure 2:**
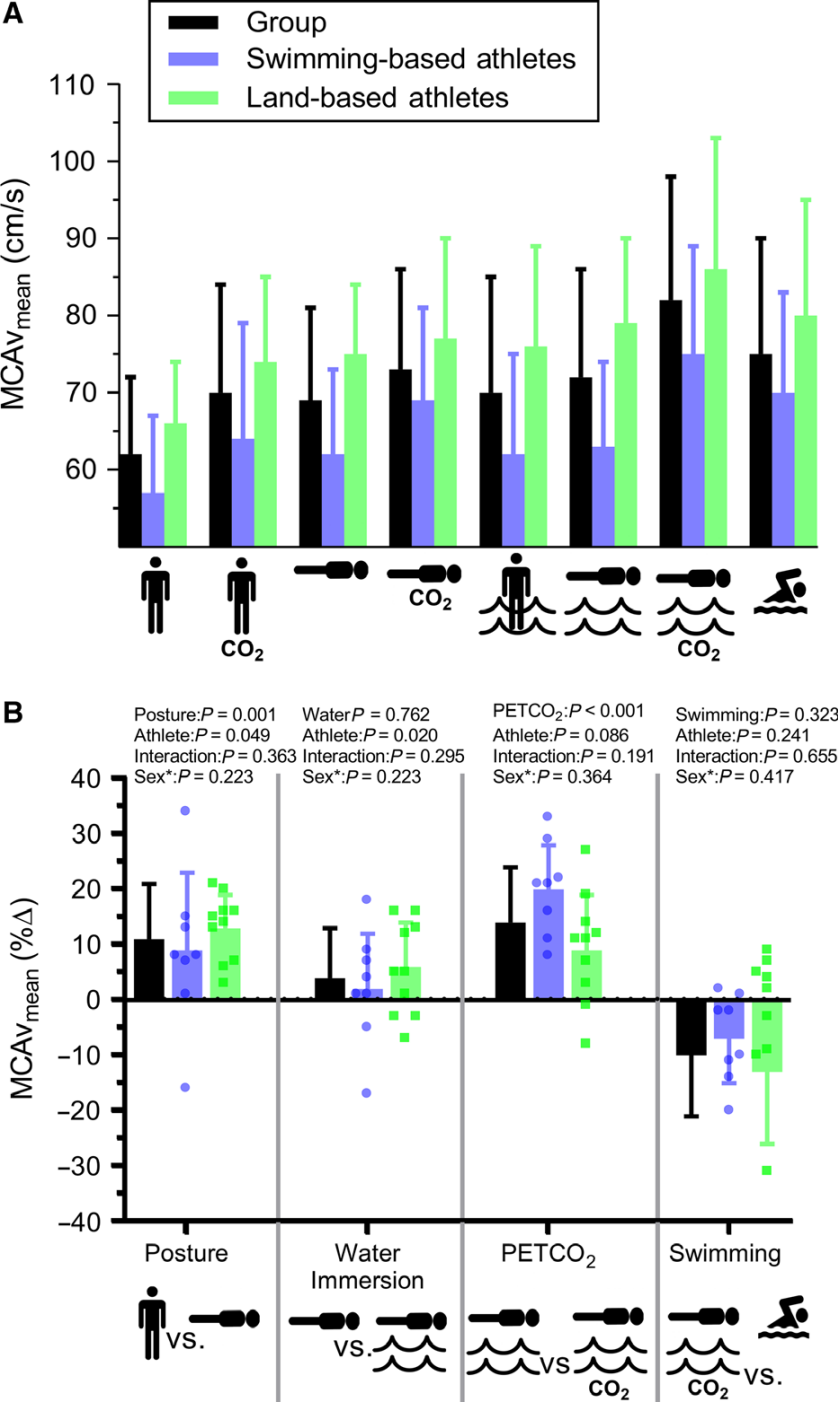
Characterization of MCAv_mean_ responses to changes in posture, water immersion, PETCO_2_, and swimming. Each comparison represents a separate ANCOVA analysis (run on absolute values; panel B). *Sex was included within each ANCOVA as a covariate. MCAv_,_ mean middle cerebral artery blood velocity; PETCO_2,_ pressures of end‐tidal CO_2_.

Swimmers had an overall ~17% lower MCAv_mean_ during normocapnic conditions (group effect: *P* ≤ 0.049, ηp^2^ ≥ 0.24, Fig. 2B), but their hypercapnic reactivity was similar to that of land‐based athletes (*P* = 0.21). Furthermore, no differences were evident for MAP, systolic or diastolic BPs, or HR (all *P* ≥ 0.66). Subsequent ultrasound data indicated no difference between ICA diameter (4.9 ± 0.5 vs. 4.9 ± 0.6 cm, *P* = 0.99) or velocity (39.8 ± 8.7 vs. 39.6 ± 10.0 cm/s, *P* = 0.97) between land‐based athletes (*N* = 8) and swimmers (*N* = 5). However, MCAv_mean_ in a matched posture (i.e., prone) for the subset of participants who underwent ultrasound remained significantly lower in swimmers (i.e., 77 ± 9 vs. 59 ± 7 cm/s, *P* < 0.01).

### Delineation of swimming related effects on MCAv_mean_

After controlling for sex, the two groups showed similar MCAv_mean_ responses to changes in posture, water immersion, and alterations in PETCO_2_ (interactions: *P* ≥ 0.19) (Fig. 2). While on land, a prone posture increased MCAv_mean_ by 11% (*P* < 0.01 vs. upright posture, ηp^2^ = 0.54). Water immersion, while in a prone posture, did not reliably change MCAv_mean_ (*P* = 0.76), but immersion in an upright posture increased MCAv_mean_ by 12% (*P* < 0.01 vs. upright on land, ηp^2^ = 0.62).

Breast‐stroke swimming at 50% HRR, compared to upright posture on land, increased HR by 51 ± 21 bpm, MCAv_mean_ by 14 ± 9 cm/s, and PETCO_2_ by 8 ± 4 mm Hg (all *P* < 0.01) (Table 2 and 3), regardless of group. The change in PETCO_2_ was independently associated with a 14% increase of MCAv_mean_ (*P* < 0.01, ηp^2^ = 0.61). Hypercapnic reactivity in a prone posture was not different between land and water (*P* = 0.49), or an upright posture (*P* = 0.97). Lastly, the motor involvement from head‐up, breast‐stroke swimming had no significant influence on MCAv_mean_ (*P* = 0.32), relative to the posture‐, PETCO_2_‐, and immersion‐ matched condition.

**Table 2:** Combined and group means (±SD) of cerebrovascular and respiratory measures during conditions on land and immersed in water.

Swimmers, *n* = 8 Land athletes, *n* = 10	Land				Water			
	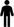	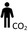	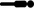	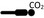	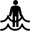	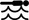	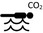	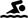
	Upright	Upright + CO_2_	Prone	Prone + CO_2_	Upright	Prone	Prone + CO_2_	Swimming
CVC (cm/s / mm Hg)								
Combined mean	0.72 ± 0.17	0.79 ± 0.21	0.85 ± 0.21^a^	0.91 ± 0.22	0.77 ± 0.19	0.84 ± 0.20	0.92 ± 0.25^b^	NA
Swimmers	0.67 ± 0.14	0.74 ± 0.22	0.76 ± 0.21	0.86 ± 0.20	0.67 ± 0.21	0.74 ± 0.19	0.89 ± 0.26	
Land‐based	0.77 ± 0.20	0.83 ± 0.22	0.92 ± 0.21	0.95 ± 0.24	0.85 ± 0.16	0.92 ± 0.18	0.94 ± 0.29	
MCAv pulsatility (AU)								
Combined mean	0.83 ± 0.15	0.77 ± 0.15	0.93 ± 0.18^a^	0.84 ± 0.12	0.92 ± 0.14	0.92 ± 0.12	0.77 ± 0.09^b^	1.4 ± 0.29^c^
Swimmers	0.78 ± 0.13	0.71 ± 0.14	0.91 ± 0.10	0.79 ± 0.15	0.91 ± 0.15	0.92 ± 0.10	0.74 ± 0.10	1.35 ± 0.31
Land‐based	0.87 ± 0.17	0.81 ± 0.13	0.94 ± 0.13	0.87 ± 0.09	0.92 ± 0.15	0.92 ± 0.14	0.80 ± 0.10	1.45 ± 0.27
PETCO_2_ (mm Hg)								
Combined mean	31 ± 3	40 ± 4	35 ± 4^a^	40 ± 4	32 ± 3	33 ± 4^d^	40 ± 4^b^	39 ± 4^c^
Swimmers	32 ± 3	42 ± 4	35 ± 4	41 ± 5	31 ± 4	32 ± 6	41 ± 5	41 ± 4
Land‐based	31 ± 3	39 ± 3	35 ± 4	39 ± 3	32 ± 3	33 ± 3	39 ± 3	38 ± 4
CVR_CO2_ (cm/s/mm Hg)								
Combined mean	NA	0.86 ± 0.72	NA	1.20 ± 1.73	NA	NA	1.44 ± 0.99	NA
Swimmers		0.77 ± 1.07		1.60 ± 1.97			1.36 ± 0.41	
Land‐based		0.92 ± 0.48		0.88 ± 1.72			1.50 ± 1.37	

PETCO_2_ values for ‘Swimming’ were analyzed from familiarization sessions during 50% heart rate range breast‐stroke swim. NA, Not Applicable as measurements were not taken during these times. CVC, cerebrovascular conductance; MCAv_,_ middle cerebral artery blood velocity; PETCO_2,_ pressures of end‐tidal CO_2_; CVR_CO2_, cerebrovascular hypercapnic reactivity.

a *P* ≤ 0.007 versus upright land.

b *P* ≤ 0.012 versus prone water.

c *P* ≤ 0.007 versus prone water + CO_2_.

d *P* ≤ 0.001 versus prone land.

**Table 3:** Combined and group means (± SD) of cardiovascular measures during conditions on land and immersed in water.

Swimmers, *n* = 8 Land‐based athletes, *n* = 10	Land				Water			
	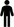	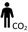	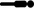	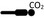	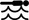	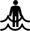	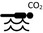	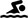
	Upright	Upright + CO_2_	Prone	Prone + CO_2_	Upright	Prone	Prone + CO_2_	Swimming
MAP (mm Hg)								
Combined mean	88 ± 11	90 ± 10	83 ± 10^a^	82 ± 10	93 ± 12	86 ± 10	90 ± 10	NA
Swimmers	87 ± 10	88 ± 8	83 ± 9	81 ± 8	95 ± 14	86 ± 11	88 ± 11	
Land‐based	88 ± 13	92 ± 12	83 ± 12	82 ± 12	91 ± 11	86 ± 11	92 ± 10	
Systolic BP (mm Hg)								
Combined mean	115 ± 13	117 ± 13	119 ± 14	117 ± 12	129 ± 12	122 ± 15	129 ± 15	NA
Swimmers	116 ± 13	116 ± 12	120 ± 15	117 ± 11	130 ± 14	127 ± 19	132 ± 21	
Land‐based	114 ± 14	117 ± 14	119 ± 15	117 ± 14	128 ± 12	119 ± 13	126 ± 12	
Diastolic BP (mm Hg)								
Combined mean	74 ± 11	77 ± 10	66 ± 10^a^	64 ± 10	77 ± 12	68 ± 11	71 ± 12	NA
Swimmers	72 ± 10	74 ± 8	67 ± 11	63 ± 8	77 ± 14	68 ± 9	66 ± 11	
Land‐based	76 ± 12	79 ± 12	65 ± 12	64 ± 11	77 ± 11	70 ± 12	75 ± 11	
HR (bpm)								
Combined mean	88 ± 16	86 ± 14	62 ± 8^a^	62 ± 8	59 ± 9	62 ± 9	68 ± 11^b^	140 ± 15^c^
Swimmers	86 ± 12	85 ± 15	63 ± 5	60 ± 8	57 ± 6	61 ± 7	67 ± 5	137 ± 8
Land‐based	89 ± 17	87 ± 13	62 ± 10	63 ± 10	60 ± 12	63 ± 11	68 ± 15	142 ± 19

MAP, mean arterial blood pressure; BP, blood pressure; HR, heart rate; NA, not applicable as measurements were not taken during these times.

a *P* ≤ 0.007 versus upright land.

b *P* ≤ 0.012 versus prone water.

c *P* ≤ 0.007 versus prone water + CO_2_.

### Cardiovascular outcomes

Prone posture decreased MAP by 5% (*P* = 0.01 vs upright land, ηp^2^ = 0.38), corresponding to a significant decrease in diastolic BP (11%; *P* < 0.01, ηp^2^ = 0.49) and HR (20%; *P* < 0.01, ηp^2^ = 0.75) (Table 3). Water immersion tended to increase MAP (*P* ≥ 0.06) but did not change HR (*P* = 0.99). Hypercapnia (+8 mm Hg) during prone‐posture water immersion significantly increased HR, by 9% (*P* < 0.01), but not MAP (*P* = 0.78). The motor involvement of swimming *per se* increased HR by 112% compared to a posture‐, PETCO_2_‐, and immersion‐ matched condition (*P* < 0.01, ηp^2^ = 0.97).

### Cognition

**Figure 3:**
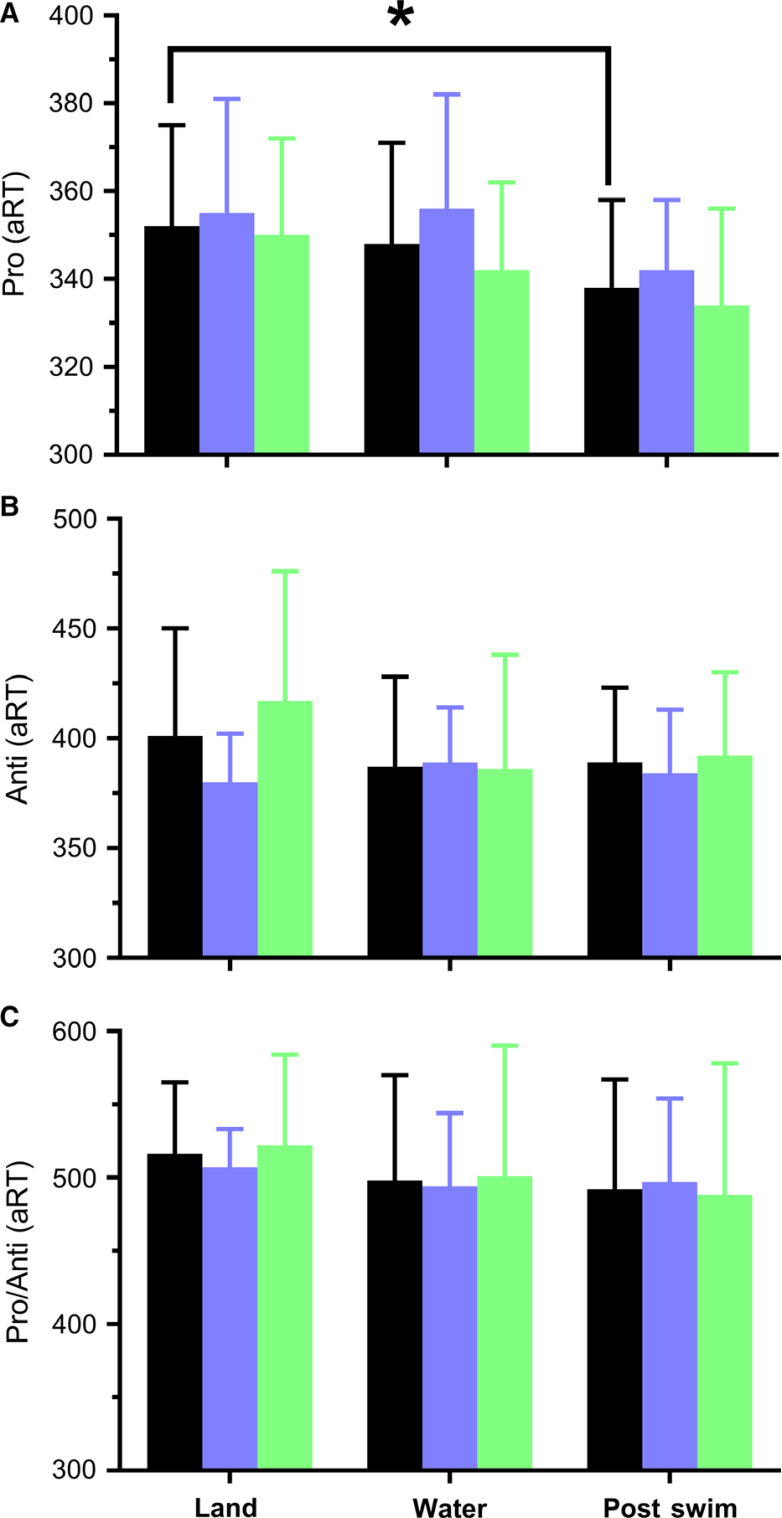
Grouped (black), swimmers (blue) and land‐based (green) athletes’ accuracy‐adjusted response time (aRT) to Pro (visuomotor speed; panel A), Anti (inhibitory control; panel B), and Pro/Anti (mental switching ability; panel C) tasks on land, in water, and after a 20‐min moderate‐intensity swim. *P = 0.01.

**Figure 4:**
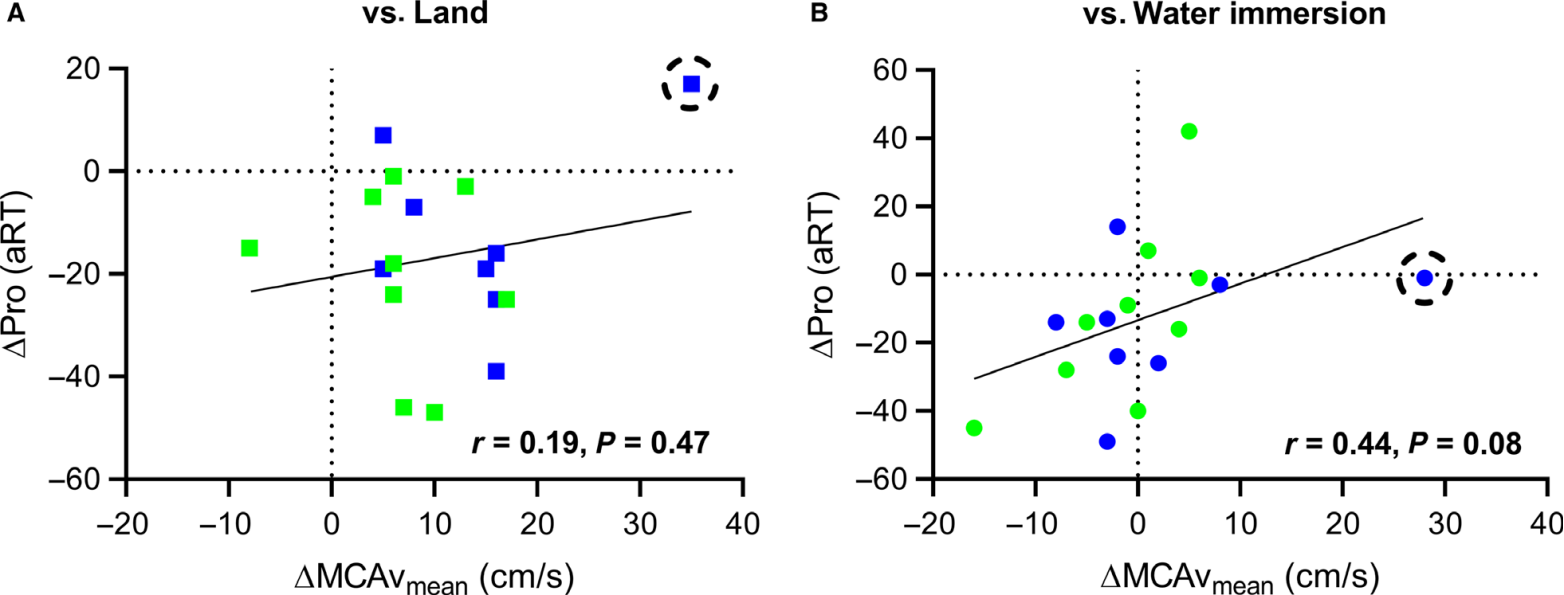
Post 20‐min breast‐stroke swim mean middle cerebral artery blood velocity (MCAv_mean_) associated with visuomotor (Pro) accuracy‐adjusted reaction time (aRT) for both swimmers (blue) and land‐based athletes (green) compared to land (panel A) and water immersion (panel B) conditions. Correlations are strengthened with the exclusion of an outlier (circled data point; *r* = −0.27, *P* = 0.31 vs. Land; *r* = 0.53, *P* = 0.03 vs. Water Immersion).

Whereas water immersion increased MCAv_mean_ in an upright posture it did not reliably alter cognition, regardless of athlete group or cognitive test (all *P* ≥ 0.15; Fig. 3). Twenty minutes of mild‐intensity swimming improved subsequent Pro trial aRT by 4% (*P* = 0.01, ηp^2^ = 0.36), independent of athlete type (interaction: *P* = 0.75). This improvement was attributable to a 3% faster reaction time (15 ± 20 ms) without compromising accuracy (which was 100%). No further effects of swimming were evident for Anti or Pro/Anti trials (all *P* ≥ 0.21). The improvement in performance of Pro trials tended to be associated (*r* = 0.44; *P* = 0.08) with decreases of MCAv_mean_ after a 20‐min swim (Fig. 4B) in both males (*r* = 0.46; *P* = 0.13) and females (*r* = 0.64; *P* = 0.09), when compared to a similar water‐immersed environment. This correlation weakened when compared to a land environment (*r* = 0.19, *P* = 0.47; Fig. 4A).

## Discussion

This study was the first to delineate the separated acute and chronic (i.e., training‐related) effects of swimming on MCAv_mean_. The 4 novel findings were that: (1) MCAv_mean_ increases during moderate‐intensity, breast‐stroke swimming mainly due to changes in posture and PETCO_2_; (2) water immersion in a prone posture does not elicit hydrostatic effects on MCAv_mean_; (3) improved cognitive performance (by virtue of aRT) tended to be associated with decreases in MCAv_mean_ after 20 min of moderate‐intensity swimming compared to a similar water‐immersed environment, and (4) swimmers showed lower MCAv_mean_ than land‐based athletes during resting conditions.

Uniquely, the current study delineated the factors behind changes in MCAv_mean_ during breast‐stroke swimming. Previous studies have illustrated that *upright* aquatic exercise (i.e., walking/running and box stepping) increases MCAv_mean_ across a range of intensities (Pugh et al., 2014; Parfitt et al., 2017), which has been contributed, in part, to changes in PETCO_2_ (Pugh et al., 2014). Furthermore, we illustrated that swimming shows a similar MCAv_mean_ response as for other dynamic large muscle‐group exercise (unpublished data from our lab). Although our data support the contribution of increased PETCO_2_, we also illustrated the large contribution of posture, and minimal contributions of motor involvement and water immersion per se.

The cerebrovasculature is very reactive to changes in PETCO_2_. We clamped PETCO_2_ at rest to match the values of each participant’s natural response to 50% HRR exercise (i.e., +8 ± 4 mm Hg vs. upright land). This allowed for an individual‐specific comparison between swimming and the direct effect of exercise‐related PETCO_2_. Compared to previous literature (Sackett et al., 2018), our participants did not appear to retain excess CO_2_ (>2 mm Hg) during water immersion, in either prone or upright postures (Table 2).

Posture is also a large contributor to changes in MCAv_mean_ between land‐based and swimming exercise. Significant differences in MCAv_mean_ between upright and prone postures on land reveal the hemodynamic adjustments necessary for maintaining cerebral perfusion. As compared to head up tilt, supine posture is associated with greater central venous pressure and stroke volume (resulting in greater systolic pressure) and baroreflex mediated sympathetic withdrawal and parasympathetic activation (thereby decreasing diastolic BP and HR; Shoemaker et al., 1999; Fenton et al., 2000; Van Lieshout et al., 2003). Our data show that on average, systolic BP tended to increase by 4 mm Hg (*P* = 0.05) and diastolic decreased by 8 mm Hg (*P* < 0.01 vs. upright land; Table 3) during prone posture. HR also decreased by 26 ± 15 bpm (*P* < 0.01 vs. upright land). Together, this supports a rise in MCAv_mean_ by virtue of increased stroke volume (i.e., potential Windkessel effect; Cooke, 2011) and potentially decreased sympathetic activity. Our data also illustrate that PETCO_2_ was increased during prone versus upright posture (4 ± 2 mm Hg; *P* < 0.01). This is consistent with previous literature, and likely due to a combination of improved ventilation‐perfusion matching and the posture‐induced excretion of venous CO_2_ stores from upright posture (Serrador et al., 2006).

Water immersion causes further cardiac adjustments. For example, water immersion in an upright posture decreased HR by 29 ± 17 bpm and increased MAP by 5 ± 13 mm Hg (Table 3), supporting previous literature (Carter et al., 2014). Water immersion in an upright posture introduces a hydrostatic gradient, causing the centralized redistribution of blood volume (Pendergast et al., 2011). In accordance with the Frank‐Starling mechanism, this would increase cardiac preload and stroke volume, and thus increase MAP. In the current study, water immersion while in a *prone* posture did not elicit a significant change in MCAv_mean_, HR, or MAP (*P* ≥ 0.06 vs. prone land). The diving reflex may have also been involved due to partial face immersion and momentary apnea. This is comparable to previous findings by Saito et al. (2014) that showed minimal changes in HR and BP with *supine* water immersion. The lack of change is likely due to the elimination of any orthostatic hypotension and decrease in hydrostatic pressure (Pendergast et al., 2011).

The cognitive benefit of exercise is well known. A recent meta‐analysis of 79 studies illustrated the positive benefit of exercise on cognition measured during, immediately, and delayed (11–20 min) after exercise (Chang et al., 2012). In the present study, both swim‐trained and land‐trained athletes had better performance in a visuomotor task after a moderate‐intensity, 20‐min swim compared to their baseline (i.e., land) performance. Therefore, our results support swimming as a mode of exercise that also improves aspects of cognitive functioning (i.e., visuomotor processing) in young adults. Our findings are consistent with previous studies that show exercise‐related improvement of information‐processing, mathematic, executive functioning and short‐term memory performance (Chang et al., 2012). A potential limitation to this finding was that the environment was not matched between conditions (i.e., land vs. water). However, when comparing both water‐immersed pre‐ vs. post‐swim, there is a trend toward significance (*P* = 0.07), as evident by a large effect size (ηp^2^ = 0.20). Furthermore, there was only an insignificant and small effect on Pro aRT between upright land and water conditions (*P* = 0.68, ηp^2^ = 0.01).

Cognition was not reliably altered with water immersion, despite most participants showing an increase of MCAv_mean_. This illustrates that acute and passive increases in MCAv_mean_ are not solely or immediately related to cognitive improvement. However, our results showed that a decrease in MCAv_mean_ after exercise associated with better cognitive performance (Fig. 4) when compared to a water‐immersed condition. The drop in MCAv_mean_ after exercise could be explained by a drop in MAP or, more likely, a larger lumen diameter in the MCA from an exercise and shear stress‐induced rise of core temperature (Padilla et al., 2011; Carter et al., 2016). Another possibility for the drop in MCAv_mean_ after exercise could also be related to the increased PETCO_2_. For example, Hoiland et al. (2017) illustrated that hypercapnia may cause shear‐mediated vasodilation in the ICA. Although MCAv_mean_ was associated with cognitive performance acutely after exercise, it is possible that the two are unrelated during swimming. This would support previous research wherein the correlation between MCAv_mean_ and cognition became uncoupled during exercise (Lucas et al., 2012). Because cognition was measured immediately (<2 min) after exercise in the current study, it remains possible that blood flow was displaced to the muscles and skin, and the improvement in cognition was caused by the exercise‐induced increase of regional cerebral metabolism (Ide and Secher, 2000). However, MCAv_mean_ is only an index of total flow. Thus, it is possible that global cerebral flow was increased after exercise, with velocity decreasing due to an increase in vessel lumen diameter. Exercise‐induced vasoconstriction of the MCA is also a possibility. For example, Verbree et al. (2017) reported a 2% decrease in MCA vessel cross‐sectional area after rhythmic handgrip exercise. Furthermore, Hellstrom et al. (1996) illustrated that total blood flow in the carotid artery increased after exercise, without a significant change in diameter. Therefore, the relation between CBV and cognition is complex, and not easily explained or mediated by isolated variables. Regardless, acute increases in cerebral perfusion (i.e., during upright water‐immersion) did not measurably improve cognitive performance.

Lastly, swimmers had significantly lower MCAv_mean_ during normocapnic conditions, despite similar MAP, HR, and PETCO_2_. This could reflect sample selection error or a real difference. The physical characteristics were statistically similar between groups; however, the cohort of swimmers had fewer females. It is well established that females have higher MCAv_mean_ than males (e.g., Peltonen et al., 2015). To account for the sex‐proportion differences between groups, and any influence it may have on MCAv_mean_, our statistical analysis included sex as a covariate. It is plausible, then, that swimmers may have a larger cross‐sectional area in their MCA than land‐based athletes, perhaps as a result of their large durations of training and the exercise‐associated increase in antegrade shear stress. Our results indicate no difference between groups in ICA diameter. It is worth considering that males have larger cerebral (Müller et al., 1991; Shatri et al., 2017) and carotid arterial diameters (Krejza et al., 2006). Thus, we cannot discount the potential sex‐effect on vessel diameter despite matching the number of male ICA measures (*N* = 3) in each group. Due to the lack of difference in ICA measures, and the unknown MCA diameters in swimmers and land‐based athletes, it is difficult to conclude swimmers have any unique vascular adaptations. Swimmers experience several other differences in their training physiology, e.g., fluid regulatory stimuli, preload, afterload and inotropic effects, and neural afferent and efferent loading distributions. Although previous research supports no difference in arterial stiffness between swimmers and land‐based athletes (Nishiwaki et al., 2017), swimmers appear to have lower blood volume and total hemoglobin mass compared to land‐trained athletes (Heinicke et al., 2001). This is likely because water immersion minimizes the effect of exercise on plasma volume expansion (Boning et al., 1988). Be that as it may, a recent meta‐analysis (Lahart and Metsios, 2018) illustrated that swim training has similar anthropometric, blood biomarker, resting cardiovascular and vascular, muscular strength, and cardiorespiratory outcomes to other modes of aerobic training (i.e., cycling and running) in healthy or clinical populations.

The study has limitations. Due to the aquatic and dynamic environment utilized, we were unable to measure MAP on a beat‐by‐beat basis or during exercise. However, it has been previously shown that MAP has a greater increase during water‐immersed upright exercise compared to that which occurs on land (Pugh et al., 2014). Furthermore, we only report data from one swimming style (i.e., breast‐stroke). Pilot testing revealed that maintaining MCAv signal integrity with front crawl proved too difficult. However, breast‐stroke is a legitimate form of swimming – accessible to people of many ages and physical abilities. Furthermore, the head‐out nature of this swimming stroke may involve slightly different cardiovascular responses than traditional submersed strokes (e.g., due to influences of the diving reflex and cranial pressures). In addition, this study was based on the usual assumption that the hypercapnic stimulus increased flow by dilation of downstream vessels without a meaningful increase in MCA diameter. Recent research supports dilation of the MCA (and other subcortical vessels) during hypercapnia of 46–48 mm Hg (Coverdale et al., 2014; Al‐Khazraji et al., 20199). However, our participants did not exceed 45 mm Hg at any point. Thus, it is possible that our data may underestimate a change in total flow, but this would not invalidate the present findings or implications discussed above. The results cannot be transferred to older or clinical populations, or to the posterior cerebral circulation. The current study did not have a time‐control for cognitive measures. However, the finding that swimming improved cognitive performance on Pro aRT is consistent with previous literature on exercise acutely benefiting cognition. Lastly, although menstrual cycle was not strictly controlled, all but one participant was in luteal phase. One female land‐based athlete reported being on day 10 of her menstrual cycle (follicular phase) and was not an outlier in terms of cerebrovascular or cognitive function.

## Conclusion

Breast‐stroke swimming increased MCAv_mean_ mostly due to postural and PETCO_2_ effects, with minimal contributions from water immersion and motor activity. Twenty minutes of moderate‐intensity swimming improved cognitive performance. Therefore, if swimming elicits cerebrovascular adaptation, as occurs in other modes of aerobic exercise, posture and hypercapnic effects are possible mediators. Furthermore, the acute benefits of swimming for cognitive performance shown here imply that regular swimming may confer chronic cognitive health benefits. This is important since swimming is an accessible form of exercise and has the potential to be beneficial for a wide range of people.

## Conflict of Interest

We have no disclosures or conflicts of interest to report.
